# Prevalence of Chromosomal Abnormalities in Iranian Patients with Infertility

**DOI:** 10.34172/aim.2023.17

**Published:** 2023-02-01

**Authors:** Saima Abbaspour, Alireza Isazadeh, Matin Heidari, Masoud Heidari, Saba Hajazimian, Morteza Soleyman-Nejad, Mohammad Hossein Taskhiri, Manzar Bolhassani, Amir Hossein Ebrahimi, Parvaneh Keshavarz, Zahra Shiri, Mansour Heidari

**Affiliations:** ^1^Cellular and Molecular Research Center, Faculty of Medicine, Guilan University of Medical Sciences, Rasht, Iran; ^2^Immunology Research Center, Tabriz University of Medical Sciences, Tabriz, Iran; ^3^Ariagene Medical Genetics Laboratory, Qom, Iran; ^4^Department of Animal Biology, Faculty of Natural Sciences, University of Tabriz, Tabriz, Iran; ^5^Department of Medical Genetics, Tehran University of Medical Sciences (TUMS), Tehran, Iran

**Keywords:** Chromosomal abnormalities, Cytogenetics, Infertility, Karyotyping

## Abstract

**Background:**

The numerical and structural abnormalities of chromosomes are the most common cause of infertility. Here, we evaluated the prevalence and types of chromosomal abnormalities in Iranian infertile patients.

**Methods:**

We enrolled 1750 couples of reproductive age with infertility, who referred to infertility clinics in Tehran during 2014- 2019, in order to perform chromosomal analysis. Peripheral blood samples were obtained from all couples and chromosomal abnormalities were evaluated by G-banded metaphase karyotyping. In some cases, the detected abnormalities were confirmed using fluorescence *in-situ* hybridization (FISH).

**Results:**

We detected various chromosomal abnormalities in 114/3500 (3.257%) patients with infertility. The prevalence of chromosomal abnormalities was 44/114 (38.596%) among infertile females and 70/114 (61.403%) among infertile males. Structural chromosomal abnormalities were found in 27/1750 infertile females and 35/1750 infertile males. Numerical chromosomal abnormalities were found in 17/1750 of females and 35/1750 of males. The 45, XY, rob (13;14) (p10q10) translocation and Klinefelter syndrome (47, XXY) were the most common structural and numerical chromosomal abnormalities in the Iranian infertile patients, respectively.

**Conclusion:**

In general, we found a high prevalence of chromosomal abnormalities in Iranian patients with reproductive problems. Our study highlights the importance of cytogenetic studies in infertile patients before starting infertility treatments approaches.

## Introduction

 Infertility is described as the incapacity to conceive naturally after one year of unprotected sexual intercourse.^1^ This condition is one of the common health problems and involves large number of couples worldwide. The epidemiological data demonstrates that 15.0% of the Iranian population are unable to conceive naturally during the first year of marriage.^2^ Most infertile couples will conceive spontaneously after the first year or respond to treatment, so that only 5.0% of the population remain unable to conceive.^3^ Various factors including genetics, infections, endocrine issues, lifestyle, and environment are involved in the infertility of males and females.^4-8^

 Chromosomal abnormalities are the most common causes of genetic defects, and are known as an important cause of reproductive problems, spontaneous abortion, and fetal death.^8^ Various chromosomal variants and major chromosomal abnormalities have been found in 1.3-15.0% of couples with reproductive problems.^9,10^ The prevalence of chromosomal abnormalities in infertile males is 1.1-7.2%,^11,12^ whereas in infertile females, it is 10.0%.^13,14^ Structural rearrangements (inversions) and sex chromosomal mosaicism are reported as the most frequent chromosomal abnormalities in individuals with reproductive problems.^15^ Moreover, balanced and reciprocal translocations are common structural rearrangements in populations with infertility.^16^

 Due to the high prevalence and remarkable effects of chromosomal abnormalities in human infertility, the aim of the present study was to investigate the prevalence of chromosomal abnormalities in an Iranian population with reproductive problems.

## Materials and Methods

###  Study Subjects

 In the present study, we enrolled 1750 couples with infertility (1750 women and 1750 men), who referred to infertility clinics (Tehran, Iran) from January 2014 until January 2019. Patients with diagnosed causes of infertility, other than chromosomal abnormalities, were excluded from our study. Genetic counselling of the studied infertile patients included inheritance risk of chromosomal abnormalities, spontaneous abortion, congenital anomalies, and fetal death. All patients were informed about the study and an informed consent was signed according to the ethical standards of the Declaration of Helsinki. The study was performed with the approval of the Institutional Review Board (IRB) of Tehran University of Medical Sciences.

###  Chromosome Analysis

 Peripheral blood samples (5 mL) of the studied patients were collected and added to a heparinized tube. Blood sample culture was performed using the RPMI-1640 culture medium containing fetal bovine serum (FBS), phytohaemagglutinin (PHA), and penicillin-streptomycin (Pen/Str). Cytogenetic analysis was conducted on Giemsa-Trypsin-Giemsa (GTG)-banded metaphase chromosomes with banding resolution of 450-550 bands per patients. Various chromosomal polymorphisms, such as pericentric inversion in the short arm of chromosome 9, were considered normal variants. In the mosaicism cases, 50–100 additional metaphases were analyzed to detect the percentage of mosaicism. The chromosomal abnormalities were observed and detected using computerized Karyotyper (Cytovision 2.7). In addition, fluorescence *in-situ* hybridization (FISH) with designed probes was performed to assess the level of mosaicism or confirm the type of abnormality in 198 cases (25). The analyzed karyotype was reported according to the International System for Human Cytogenetic Nomenclature.

## Results

###  Clinical Information of Patients

 In the present study, we enrolled 1750 infertile couples (31 ± 1.8 years old). The demographic characteristics and general features of the infertile patients are presented in Table 1.

**Table 1:** Demographic Characteristics and General Features of Patients with Infertility

**Characteristic**	**Women (n=1750)**	**Men (n=1750)**
Age (y)	27.18 ± 2.76	35.84 ± 1.19
Duration of infertility (y)	5.56 ± 2.24	4.78 ± 2.81
Body mass index (kg/m^2^)	25.56 ± 3.53	23.15 ± 2.25
Age at menarche (year)	13.11 ± 1.22	-
Tobacco smoking (%)	114 (6.51%)	611 (34.91%)
Alcohol drinking (%)	86 (4.91%)	501 (28.62%)
Genetic background (%)	41 (2.34%)	38 (2.17%)

###  Normal Chromosomal Variants

 The karyotypes of 59 (1.685%) all infertile patients were found with normal chromosomal variants, including inv(9)(p11q13), 13pstk, 14pstk, 15pstk, 22pstk. We also observed other rare normal variants in the infertile patients, including enlarged heterochromatin of the chromosome 9q and reduced length of heterochromatin in chromosome Y. The normal chromosomal variants are presented in Table 2.

**Table 2:** Normal Chromosomal Variants Observed in 3500 Patients with Infertility

**Normal Variants**	**Females (n=1750)**	**Males (n=1750)**	**Total (n=3500)**
13pstk	1 (0.057%)	1 (0.057%)	2 (0.057%)
14pstk	2 (0.114%)	1 (0.057%)	3 (0.085%)
15pstk	11 (0.628%)	13 (0.742%)	24 (0.685%)
22pstk	1 (0.057%)	3 (0.171%)	4 (0.114%)
inv(9)(p11q13)	9 (0.171%)	7 (0.400%)	16 (0.457%)
Enlarged 9q	2 (0.114%)	—	2 (0.057%)
Reduced Yq	—	8 (0.457%)	8 (0.228%)
**Total**	26 (1.485%)	33 (1.885%)	59 (1.685%)

###  Total Chromosomal Abnormalities

 The abnormal karyotype was observed in 3.257% (114 cases) of patients with infertility. The prevalence of abnormal karyotype in infertile males was 61.403% (70 cases), whereas it was 38.596% (44 cases) in infertile females. The prevalence of structural chromosomal abnormalities (62/3500) was more than the prevalence of numerical chromosomal abnormalities (52/3500) in all patients. We observed only one case with Y microdeletion, 46, XY, del(Y)(q11.2), and one case with deletion in the AZFc region. All structural and numerical chromosomal abnormalities detected in infertile patients are presented in Tables 3 and 4.

**Table 3:** Structural Chromosomal Abnormalities Observed in 3500 Patients with Infertility

**Structural Chromosomal Abnormalities**	**Karyotype **	**Males (n=1750)**	**Females (n=1750)**	**Total (n=3500)**
Duplications	46, XX, dup(18)(p11;32)	-	1 (0.057%)	1 (0.028%)
Ring chromosome	46, XX, r[18](p11q22)	1 (0.057%)	—	1 (0.028%)
Deletions	46, XY, del(Y)(q11.2)	1 (0.057%)	—	1 (0.028%)
	46, XY, del(18)(q22)	1 (0.057%)	—	1 (0.028%)
Inversion	46, XY, inv(4)(p13q13)	1 (0.057%)	—	1 (0.028%)
	46, XY, inv(2)(p24q12)	1 (0.057%)	—	1 (0.028%)
	46, XY, inv(6)(p21.2q22)	1 (0.057%)	—	1 (0.028%)
	46, XY, inv(7)(p15.1q22)	1 (0.057%)	—	1 (0.028%)
	46, XX, inv(7)(p31.1q33)	—	1 (0.057%)	1 (0.028%)
	46, XX, inv(9)(p11q13)	—	1 (0.057%)	1 (0.028%)
	46, XY, inv(1)(q13p31)	1 (0.057%)	—	1 (0.028%)
	46, XX, inv(1)(q23p13)	—	1 (0.057%)	1 (0.028%)
	46, XX, inv(8)(p22q13)	—	1 (0.057%)	1 (0.028%)
	46, XY, inv(9)(p11q12)	1 (0.057%)	—	1 (0.028%)
	46, XX, inv(9)(p11q12)	—	1 (0.057%)	1 (0.028%)
	46, XX, inv(9)(p12q13)	—	3 (0.171%)	3 (0.085%)
	46, XY, inv(9)(p12q13)	1 (0.057%)	—	1 (0.028%)
	46, XX, inv(9)(p13q34)	—	2 (0.114%)	2 (0.057%)
	46,XY,inv(9)(p13q34)	4 (0.228%)	—	4 (0.114%)
Translocations	45, XY, rob(14;21)(p10q10)	1 (0.057%)	—	1 (0.028%)
	45, XX, rob(13;14)(p10q10)	—	2 (0.114%)	2 (0.057%)
	45, XY, rob(13;14)(p10q10)	7 (0.400%)	—	7 (0.200%)
	46, XY, t(1;13)(p32.2q34)	1 (0.057%)	—	1 (0.028%)
	46, XY, t(1;12)(p32.2q15)	1 (0.057%)	—	1 (0.028%)
	46, XY, t(2;8)(p37.2q11)	1 (0.057%)	—	1 (0.028%)
	46, XY, t(9;15)(p21q15)	1 (0.057%)	—	1 (0.028%)
	46, XY, t(13;14)(p21.1q32.3)	1 (0.057%)	—	1 (0.028%)
	46, XY, t(17;22)(p21.3q13.3)	1 (0.057%)	—	1 (0.028%)
	46, XX, t(1;19)(p13q13.3)	—	1 (0.057%)	1 (0.028%)
	46, XY, t(9;3)(q32q28)	1 (0.057%)	—	1 (0.028%)
	46, XX, t(2;18)(q36q24)	—	1 (0.057%)	1 (0.028%)
	46, XX, t(4;13)(p11q11)	—	4 (0.228%)	4 (0.114%)
	46, XX, t(1,14)(p22q22)	—	1 (0.057%)	1 (0.028%)
	46, XX, t(11;13)(p21q32)	—	1 (0.057%)	1 (0.028%)
	45, XY, t(11;22)(q23q11)	1 (0.057%)	—	1 (0.028%)
	45, XY, der(13;14)(q10q10)	1 (0.057%)	—	1 (0.028%)
	46, XY, t(3;7)(p27p21)	1 (0.057%)	—	1 (0.028%)
	46, XY, t(4;16)(q12p13)	1 (0.057%)	—	1 (0.028%)
	46, XY, t(1;16)(p11p11)	1 (0.057%)	—	1 (0.028%)
	46, XX, t(10;14)(q12q22)	—	1 (0.057%)	1 (0.028%)
	46, XX, t(3;9)(p13q24)	—	1 (0.057%)	1 (0.028%)
	46, XY, t(6;19)(q16q13)	—	1 (0.057%)	1 (0.028%)
	45, XY, t(6;20)(p27q11.2)	1 (0.057%)	—	1 (0.028%)
	46, XX, t(1;13)(q22q14)	—	1 (0.057%)	1 (0.028%)
	46, XX, t(3;4)(q12p15)	—	1 (0.057%)	1 (0.028%)
	46, XX, t(3;4)(q13q21)	—	1 (0.057%)	1 (0.028%)
Total		35 (2.000%)	27 (1.542%)	62 (1.771%)

**Table 4:** Numerical Chromosomal Abnormalities Observed in 3500 Patients with Infertility

**Numerical Chromosomal Abnormalities**	**Karyotype **	**Males (n=1750)**	**Females (n=1750)**	**Total (n=3500)**
Turner syndrome	45, X	—	7 (0.400%)	7 (0.200%)
Klinefelter syndrome	47, XXY	26 (1.485%)	—	26 (0.742%)
47, XYY syndrome	47, XYY	6 (0.342%)	—	6 (0.171%)
47, XXX syndrome	47, XXX	—	4 (0.228%)	4 (0.114%)
Mosaicism	45, X, [4]/46, XX[92]/47, XXX [4]	—	1 (0.057%)	1 (0.028%)
	45, X [6]/47, XXX [24], Turner	—	1 (0.057%)	1 (0.028%)
	45, X [3]/46, X, i(X)(q10)[17], Turner	—	1 (0.057%)	1 (0.028%)
	45, X, [2]/46, XX, [63]	—	1 (0.057%)	1 (0.028%)
	45, X [7]/46, XX [43]	—	1 (0.057%)	1 (0.028%)
	47, XY + mar [35]/46, XY [65]	1 (0.057%)	—	1 (0.028%)
	46, XY, t(1;2)(p11.1q11.1)[15]/46, XY [85]	1 (0.057%)	—	1 (0.028%)
Isochromosome	46, XY, i(20)(q10)	1 (0.057%)	—	1 (0.028%)
	46, X, i(X)(q10)	—	1 (0.057%)	1 (0.028%)
Total		35 (2.000%)	17 (0.971%)	52 (1.485%)

###  Structural Chromosomal Abnormalities

*Translocations:*Translocations are the most frequent type of structural chromosomal abnormalities, which were observed in 1.057% (21 males and 16 females) of patients with infertility. These abnormalities were detected in 32.456% of the all chromosomal abnormalities. Moreover, Robertsonian translocation was detected in 10 cases (8 males and 2 females).

*Inversions:* Inversions are the second most frequent structural chromosomal abnormalities, which were observed in 0.600% (11 males and 10 females) of patients with infertility. These abnormalities constituted 18.421% of all chromosomal abnormalities. All detected inversions were pericentric (21 cases), and we did not find any paracentric inversions.

*Deletions:* Deletions were found in 0.057% (2 males) of patients with infertility, which constituted 1.754% of all chromosomal abnormalities. The observed deletions occurred in the chromosomes 18 and Y of 2 males with infertility.

*Duplications:* Duplications were detected in 0.028% (one female) of patients with infertility, which constituted 0.877% of all detected chromosomal abnormalities. The observed duplication was found in the chromosome 18 of one female with infertility.

**Figure 1:**
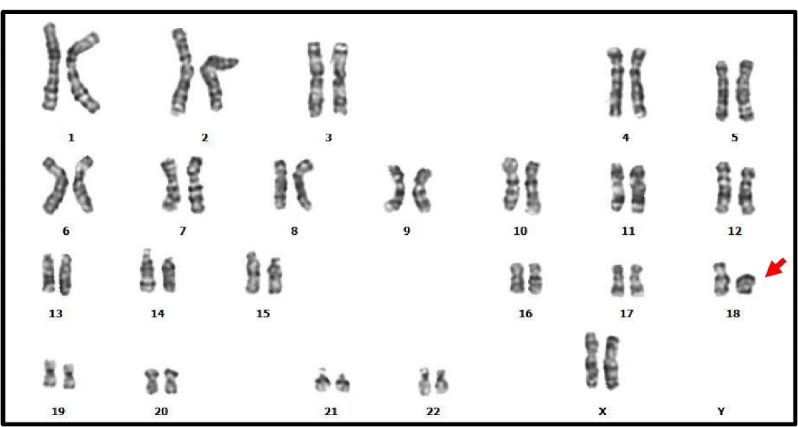
The Karyotype by Giemsa Banding of a Infertile Female with Infertility. We found a ring chromosomes: 46, XX, r[18](p11q22).

*Ring chromosome:*Ring chromosomes were observed in 0.028% (one female), which constituted 0.877% of all chromosomal abnormalities. The observed ring chromosome 18 was detected in one female with infertility (Figure 1).

###  Numerical Chromosomal Abnormalities

*Klinefelter syndrome:*The Klinefelter syndrome (47, XXY) is the most frequent type of numerical chromosomal abnormality, which was observed in 0.742% (26 males) of patients with infertility. This abnormality constituted 22.807% of all chromosomal abnormalities, and 50% of numerical chromosomal abnormalities.

*Turner syndrome:* The Turner syndrome (45, X) is the second most frequent numerical chromosomal abnormality, which was observed in 0.200 (7 females) of patients with infertility. This abnormality constituted 6.140% of all chromosomal abnormalities, and 13.461% of all numerical chromosomal abnormalities.

*47, XYY syndrome:*The 47, XYY syndrome was detected in 0.171% (6 males) of patients with infertility. This abnormality constituted 3.263% of all chromosomal abnormalities, and 11.538% of numerical chromosomal abnormalities.

*47, XXX syndrome:*The 47, XXX syndrome was detected in 0.114% (4 females) of patients with infertility. This abnormality constituted 5.508% of all chromosomal abnormalities, and 7.692% of numerical chromosomal abnormalities.

*Isochromosome:*Isochromosomes were detected in 0.057% (one male and one female). This abnormality constituted 1.754% of all chromosomal abnormalities, and 3.846% of numerical chromosomal abnormalities. The isochromosome of chromosome 20 was observed in one male and the isochromosome of chromosome X was observed in one female.

*Mosaicism:*Mosaicism was detected in 0.20% (2 males and 5 females). This abnormality constituted 5.263% of all chromosomal abnormalities, and 11.538% of numerical chromosomal abnormalities.

###  Sex Chromosome Abnormalities

 Sex chromosomal abnormalities were detected in 49 cases (42.982% of all chromosome abnormalities), consisting of 33 males (66.000%) and 17 females (34.000%). Aneuploidy of the X chromosome was observed in 43 patients, which included 11 females with karyotype 45, X and 47, XXX as well as 32 males with karyotype 47, XXY and 47, XYY. Moreover, sex chromosome mosaicism was observed in 5 females. Structural Y chromosome abnormality (deletion) was detected in one male with infertility (Tables 3 and 4).

###  Autosomal Chromosomes Abnormalities

 Autosomal chromosomal abnormalities were detected in 64 cases (56.140% of all chromosome abnormalities), consisting of 37 males (57.812%) and 27 females (42.187%). The observed autosomal abnormalities included inversion, translocations (reciprocal and Robertsonian), isochromosome, and ring chromosome (Tables 3 and 4).

## Discussion

 Infertility is a troubling and destructive event in young couples, which can be remedied by current approaches in a high proportion of patients. However, identification of the etiology is an important consideration for appropriate and effective treatment strategies. The genetical and chromosomal abnormalities are the most important and frequent risk factors in patients with infertility.^10,11^ The high prevalence of various chromosomal abnormalities suggests that the chromosomal analysis of couples can be useful in reproduction management and healthy pregnancy.^12^ Chromosomal analysis or karyotyping is an appropriate diagnostic approach that provides important genetic information from couples. Identification of chromosomal breakpoint regions is important for recognition of genes involved in molecular mechanisms underlying human reproduction, and helping with the management of pregnancy.

 The present study was designed to identify chromosomal abnormalities in Iranian men and women with infertility. We demonstrated that the prevalence of chromosomal abnormalities in Iranian infertile patients was 3.257% (61.403% in males and 38.596% in females) which is similar to other studies in different countries.^17,18^ In a study by Clementini et al, chromosomal abnormalities were observed in 82 cases (3.95%) out of 2078 patients with infertility.^18^ In another study by Pylyp et al, chromosomal abnormalities were reported in 81 cases (2.79%) out of 3414 patients with infertility.^14^ In addition, the prevalence of chromosomal abnormalities in patients with infertility ranged from 1% by Liang et al in China to 6% by Gekas et al. in France.^19,20^ Furthermore, several other studies have reported the prevalence of chromosomal abnormalities in couples with infertility in the worldwide. The prevalence of chromosomal abnormalities in infertile patients depends largely on characteristics of the studied population (Table 5).

**Table 5:** Prevalence of Chromosomal Abnormalities in Couples with Infertility in Different Studies

**Authors**	**Year**	**Region**	**Sample Size (n)**			**Chromosomal Abnormalities**		
			**Men**	**Women**	**Total**	**Men**	**Women**	**Total**
El-Dahtory et al^9^	2022	Egypt	1290	860	2150	150 (11.62%)	140 (16.27%)	290 (13.48%)
Pashaei et al^12^	2021	Iran	528	527	1055	19 (3.59%)	15 (2.84%)	34 (3.22%)
Pylyp et al^14^	2015	Ukraine	1673	1741	3414	47 (2.80%)	34 (1.95%)	81 (2.37%)
Kayed et al^15^	2006	Egypt	2650	2650	5300	138 (5.20%)	24 (0.90%)	162 (3.05%)
Serapinas et al^16^	2021	Lithuania	99	99	198	4 (4.04%)	9 (9.09%)	13 (6.56%)
Clementini et al^18^	2005	Italy	2078	2078	4156	42 (2.02%)	40 (1.92%)	82 (1.97%)

 In our study, the structural chromosomal abnormalities were more than numerical chromosomal abnormalities, constituting 54.38% of all chromosomal abnormalities in infertile patients. Among structural abnormalities, translocations were the most frequent rearrangement and were observed in 59.67% of all structural chromosomal abnormalities. All detected translocations in this study were familiar with the same breakage and reunion regions. However, a number of non-familial translocations with individual breakage and reunion regions in different families have been reported by previous studies.^21,22^ A high number of palindromic AT repeats in a chromosomal region is one of the important reasons of double-stranded DNA breaks that leads to formation of non-familial translocations through non-homologous ends binding.^21^ Inversions were the second most frequent type of chromosomal rearrangements in our study which were observed in 18.42% of infertile patients. The paracentric and pericentric inversions of chromosomes 1, 2, 4, 6, 7, 8, and 9 were detected in our study. The pericentric inversion of chromosome 9 was considered as a normal variant.^23^ However, evidence suggests that the prevalence of inversions in patients with infertility is higher than the general population.^23,24^

 Production of unbalanced gametes is the most important cause of reproductive problems in carriers of structural chromosomal rearrangements. The prevalence of unbalanced sperm in carriers of Robertsonian and reciprocal translocations ranges from 9 to 29% and 37 to 81%, respectively. However, the prevalence of unbalanced sperm in carriers of inversion ranges from 0.2 to 38% that highly depends on the size of the inverted segment.^25^ Therefore, prenatal diagnosis is an essential approach to exclude fetuses with unbalanced karyotype in carriers of chromosomal abnormalities. In addition, preimplantation genetic diagnosis is an appropriate alternative approach to invasive prenatal diagnosis in couples where pregnancy termination is not possible.^26^ In this applied method, normal embryos with balanced karyotype are transferred to the uterus, thus increasing the probability of healthy pregnancy, and significantly decreasing the risk of miscarriage and livebirth of children with unbalanced chromosomal abnormality.^27^

 In the present study, sex chromosome abnormalities comprised 42.98% of all chromosomal abnormalities in infertile patients, consisting of sex chromosomes aneuploidies (97.95%) and chromosome Y microdeletion (2.05%). Klinefelter syndrome (47, XXY) comprised 53.06% of all sex chromosome abnormalities and was the most frequent type of sex chromosome abnormality. A study by Pylyp et alreported that Klinefelter syndrome comprised 57.89% of all sex chromosome abnormalities in patients with infertility in a Ukrainian population.^14^ Klinefelter syndrome was the most common chromosome abnormality in men with oligozoospermia and azoospermia. However, the prevalence of this aneuploidy is quite variable across different populations.^18^ The second chromosomal abnormality was chromosome Y microdeletion which was observed in only one infertile male. The mosaic karyotype of sex chromosomes comprised 4.38% of all chromosomal abnormalities in patients with infertility. Sex chromosome mosaicism is normally observed in men with severe oligozoospermia.^28^

 The prevalence of sex chromosome aneuploidies in men was higher than women. In this study, we detected four women with X chromosome trisomy, seven women with X chromosome monosomy, and five women with mosaicism of the X chromosome. Women with X chromosome trisomy (47, XXX) are at high risk for premature ovarian failure. Furthermore, women with X chromosome monosomy (45, X) are rarely infertile.^29^ However, pregnancy can occur in only 2-5% of patients with mosaic Turner syndrome, and partly conserved ovarian function is observed in approximately 30% of cases.^30^

 Heterochromatin variants are commonly identified in patients with recurrent pregnancy loss, reproductive failure, and infertility. In this regard, variants of chromosomes 9 and 15 are the most frequent.^31,32^ In our study, normal chromosomal variants were observed in 1.68% of patients with infertility, with 15pstk and 9inv(9)(p11q13) being the most commonly identified chromosomal variants. In addition, heterochromatin chromosome 9qh + has been reported as the most common normal chromosomal variant.^33^ However, we did not find any infertile patients with the heterochromatin chromosome 9qh + variant. Despite the high prevalence of chromosomal normal variants in patients with infertility, the exact role or function of this abnormality remain unknown.^34,35^

 Generally, we detected 114/3500 (3.257%) chromosomal abnormalities in Iranian couples with infertility. We showed that the prevalence of autosomal and sex chromosome abnormalities was higher in males. Our study highlighted the importance of cytogenetic studies in identification of infertility etiology in infertile patients, which can lead to the use of an appropriate therapeutic approach. We suggest cytogenetic investigation in infertile couples and subsequent genetic counseling in cases with chromosomal abnormalities.
